# Cholera

**Published:** 1858-01

**Authors:** 


CHOLERA.—This terrible scourge is again upon its westward course, and has already reached the suburbs of London. The Metropolitan Board of Health has issued a circular calling attention to the various sanitary measures known to limit its spread and virulence. Its progress on the continent may be judged of from the statement that at Hamburg, "between the 29th of August and the 6th of September, there were 239 cases, of which 136 proved fatal. We have not received later accounts of the exact number of cases and deaths, but we have been informed that the mortality has been very large. Dr. Webster, in his late Northern tour, followed in the track of the pestilence from Königsberg westward. He describes the severity of the epidemic as extreme. At Upsala the University had been closed, and many of the inhabitants had fled. The Board of Health, then, is certainly not premature in issuing a circular of precautionary advice to Local Boards of Health. Cleanliness, ventilation, pure water, are the great objects—the Public Health Act, the Nuisances Removal Act, and the Common Lodging-House Act, the means of effecting them. We trust that all will join cordially in assisting the Local Boards to exercise their powers to the fullest extent, in the hope of meeting the common danger; and, as an additional precautionary measure, we trust that the most stringent instructions will be given to our Custom-House authorities to prevent a direct importation of the disease. We are perfectly aware of all the arguments against quarantine and the contagiousness of cholera; but we still think that it would be a most culpable piece of neglect and infatuation to admit cholera patients arriving from Hamburg or elsewhere to unrestricted intercourse with the inhabitants of any of our ports.—*Times and Gazette*, Oct. 10, 1857.

---

SUBJECTS IN VIENNA.—During the last fortnight the medical professors and students at the General Hospital of Vienna have been in a state of excitement and irritation. The Archbishop of Vienna not long since gave orders that all persons who died in the various Hospitals, and in the Lying-in and Foundling establishments, should be buried without either *post-mortem* examination or dissection; and the consequence of the measure is that, during the last ten or fifteen days, there has only been one subject in the great dissecting halls of the General Hospital. Vienna was proud, and had good reason to be proud, of its Medical School, but the Concordat has given into the hands of the clergy the power to ruin its reputation. According to a decree of Joseph II., the body of every person who died in the public hospital was to be opened or dissected, as the case might be; but the imperial ordinance has, *de facto*, been abrogated by the Cardinal Archbishop of Vienna.—*Times and Gazette*, Nov. 14, 1857.

---

MEDICAL STUDENTS IN LONDON.—The number of students registered at the different medical schools in London this session is 1050. This is somewhat below the usual attendance, a fact which some of the English journals account for by reference to the increasing difficulties of procuring anatomical material.

